# Early manifestations and differential gene expression associated with photoreceptor degeneration in *Prom1*-deficient retina

**DOI:** 10.1242/dmm.048962

**Published:** 2021-11-24

**Authors:** Yuka Kobayashi, Shizuka Watanabe, Agnes Lee Chen Ong, Manabu Shirai, Chiemi Yamashiro, Tadahiko Ogata, Fumiaki Higashijima, Takuya Yoshimoto, Takahide Hayano, Yoshiyuki Asai, Noriaki Sasai, Kazuhiro Kimura

**Affiliations:** 1 Department of Ophthalmology, Yamaguchi University Graduate School of Medicine, 1-1-1 Minami-kogushi, Ube 755-0046, Japan; 2 Developmental Biomedical Science, Division of Biological Sciences, Nara Institute of Science and Technology, 8916-5 Takayama-cho, Ikoma 630-0192, Japan; 3 Omics Research Center (ORC), National Cerebral and Cardiovascular Center, 6-1 Kishibe Shinmachi, Suita, Osaka 564-8565, Japan; 4 Department of Systems Bioinformatics, Yamaguchi University Graduate School of Medicine, 1-1-1 Minami-kogushi, Ube 755-0046, Japan

**Keywords:** Prominin-1, Photoreceptor, Glial cell, Retinal degeneration, Endothelin-2, Endothelin receptor antagonist

## Abstract

Retinitis pigmentosa (RP) and macular dystrophy (MD) are characterized by gradual photoreceptor death in the retina and are often associated with genetic mutations, including those in the prominin-1 (*Prom1*) gene. *Prom1*-knockout (KO) mice recapitulate key features of these diseases including light-dependent retinal degeneration and constriction of retinal blood vessels. The mechanisms underlying such degeneration have remained unclear, however. We here analysed early events associated with retinal degeneration in *Prom1*-KO mice. We found that photoreceptor cell death and glial cell activation occur between 2 and 3 weeks after birth. Whereas gene expression was not affected at 2 weeks, the expression of several genes was altered at 3 weeks in the *Prom1*-KO retina, with the expression of that for endothelin-2 (*Edn2*) being markedly upregulated. Expression of *Edn2* was also induced by light stimulation in *Prom1*-KO mice reared in the dark. Treatment with endothelin receptor antagonists attenuated photoreceptor cell death, gliosis and retinal vessel stenosis in *Prom1*-KO mice. Our findings thus reveal early manifestations of retinal degeneration in a model of RP/MD and suggest potential therapeutic agents for these diseases.

This article has an associated First Person interview with the first author of the paper.

## Abstract (precis)

**Summary:** The early manifestations of an inherited retinal disease was investigated by means of a transcriptome analysis, and a possible drug-based therapeutic approach (endothelin receptor antagonists) is suggested.

## INTRODUCTION

Both retinitis pigmentosa (RP) and macular dystrophy (MD) are inherited retinal disorders associated with progressive photoreceptor cell death (Ferrari et al., 2011). These diseases have a combined prevalence of 1 in 3000 to 4000 people worldwide. Initial symptoms include nyctalopia (night blindness) and visual field deficits, which are followed by loss of visual acuity and colour blindness and eventually by complete blindness. More than 60 genes encoding various types of protein – including membrane proteins, transcription factors, splicing regulators and enzymes related to the visual cycle – have been implicated in RP and MD (Ferrari et al., 2011). These conditions remain incurable, with effective therapeutic strategies remaining to be established, and they have profound effects on the quality of life.

The prominin-1 gene (*Prom1*, also known as *CD133* and *RP41*) encodes a pentaspan transmembrane glycoprotein that is expressed in photoreceptor cells of the retina as well as in kidney and testis (Fargeas et al., 2004). Several mutations of *PROM1* have been identified in individuals with RP or MD (Maw et al., 2000; Michaelides et al., 2010; Yang et al., 2008), with all such mutations resulting in amino acid substitutions or carboxyl-terminal truncations of the encoded protein. The mechanisms underlying RP and MD associated with *PROM1* mutations have been investigated in studies of several lines of *Prom1*-knockout (KO) mice (Dellett et al., 2015; Michaelides et al., 2010; Zacchigna et al., 2009). Although photoreceptor cells develop normally in these KO mice, they begin to degenerate after birth, resulting in a progressive loss of the outer nuclear layer (ONL) of the retina and recapitulation of the signs of RP and MD. The retinal vasculature also becomes attenuated with disease progression (Zacchigna et al., 2009). Markedly dysmorphic photoreceptors are also apparent in *Prom1*-mutant frogs (Carr et al., 2021), suggestive of a conserved role for Prom1 in photoreceptor function.

We previously showed that photoreceptor cells of the *Prom1*-KO mouse retina degenerate in response to light stimulation. Such mice reared in a completely dark setting thus manifested a marked delay in the loss of photoreceptor cells. We therefore suggested that the mutant retinal cells are hypersensitive to light stimulation and experience phototoxicity (Dellett et al., 2015). The visual cycle was also found to be impaired in the *Prom1*-KO cells, and treatment based on chemical compounds that modulate the visual cycle was found to mitigate the mutant phenotype (Dellett et al., 2015).

The Prom1 protein localizes to the connecting cilium and outer segment of both rod and cone photoreceptors (Maw et al., 2000). Ultrastructural analysis revealed the structure of the outer segment to be severely disorganized in photoreceptor cells of *Prom1*-KO mice, whereas other photoreceptor components – including the inner segment, nucleus and axon – remained largely intact (Dellett et al., 2015; Zacchigna et al., 2009). Biochemical analysis has shown that two tyrosine residues in the carboxyl-terminal region of Prom1 are phosphorylated by the tyrosine kinases Src and Fyn, although the physiological implications of such phosphorylation remain to be elucidated (Boivin et al., 2009). Prom1 has also been shown to interact with the p85 regulatory subunit of phosphatidylinositol 3-kinase (PI3K; also known as PIK3R1) and to be essential for both the self-renewal and tumorigenic capacity of glioma stem cells (Wei et al., 2013). In addition, Prom1 has been detected in cilia, which are protrusive structures at the cell membrane and key signalling hubs (Khatri et al., 2014), and to be essential for maximization of Hedgehog signalling in neural stem cells (Singer et al., 2019). We recently showed that Prom1 activates the small GTPase Rho and regulates chloride conductance triggered by intracellular calcium uptake (Hori et al., 2019). However, the mechanisms by which Prom1 prevents retinal degeneration triggered by light stimulation have remained elusive.

To characterize the role of Prom1 dysfunction in retinal degeneration and thereby to provide insight into potential treatments for *Prom1* mutation-associated RP and MD, we here investigated the initial manifestations of such degeneration. We analysed *Prom1* expression as well as the ONL transition in *Prom1*-KO mice. We then performed a high-throughput expression analysis to identify genes responsible for degeneration of the Prom1-deficient retina, and we focused on one such gene, endothelin-2 (*Edn2*), which encodes a vasoconstrictor peptide (ET-2) (Yanagisawa et al., 1988), for which expression was aberrantly induced. We further found that a chemical treatment targeted to endothelin signalling mitigated the deterioration of retinal structure and function in *Prom1*-KO mice, suggesting a new therapeutic target for RP and MD.

## RESULTS

### *Prom1* is expressed in the retina from perinatal to adult stages

**Fig. 1.**
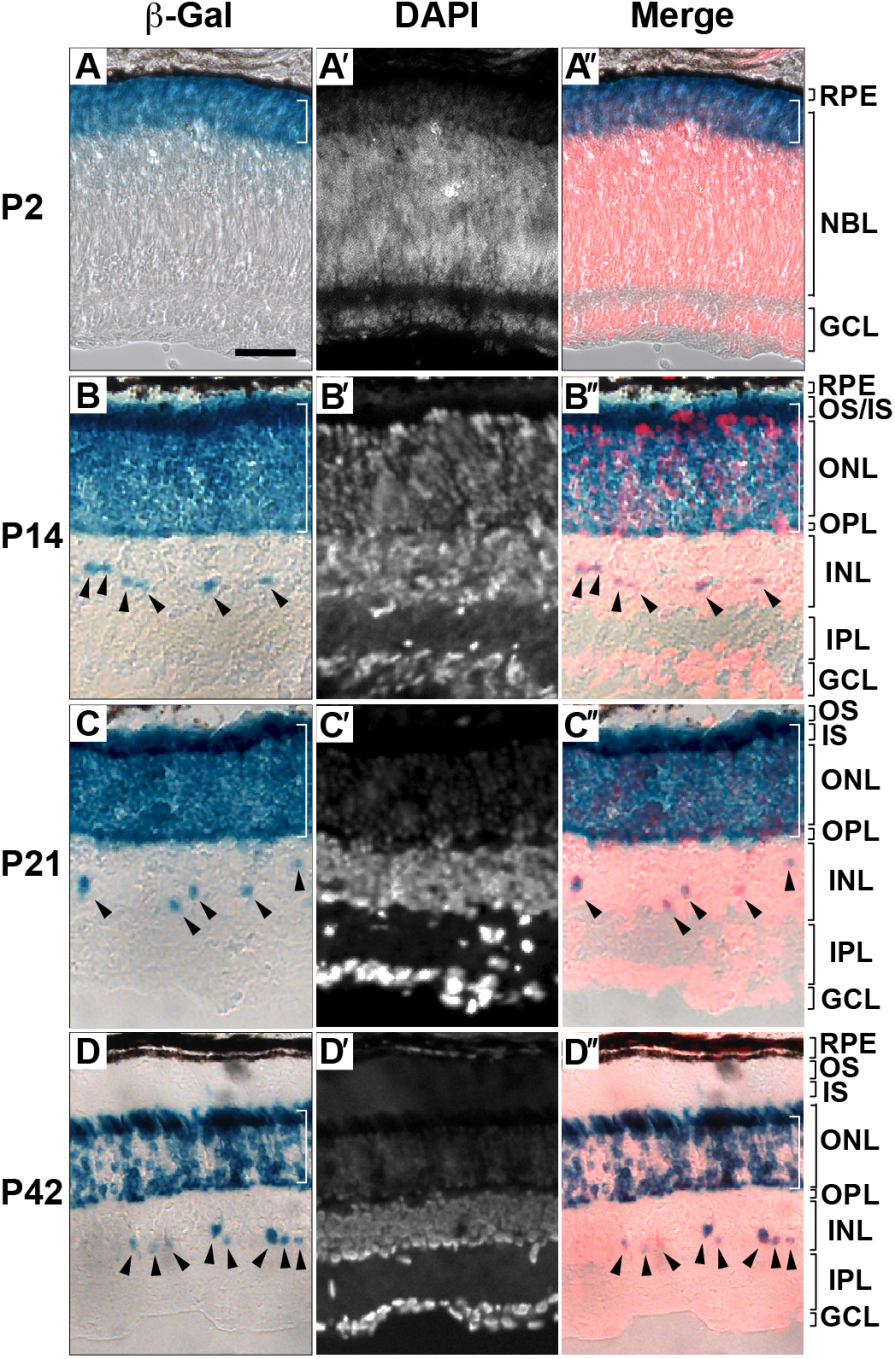
***Prom1* is expressed in the ONL of the retina from perinatal to adult stages.** (A-D″) The retina of heterozygous *Prom1* mutant mice at P2 (A-A″), P14 (B-B″), P21 (C-C″) and P42 (D-D″) was subjected to staining of β-gal activity (A,B,C,D) as well as to staining of nuclei with DAPI (white, A′,B′,C′,D′; red, A″,B″,C″,D″). Merged images are shown (A″,B″,C″,D″). Expression in the outer layers (white brackets in A,A″,B,B″,C,C″,D,D″) and in the inner layer (arrowheads in B,B″,C,C″,D,D″) is indicated. Data are representative of three retinas at each age. Scale bar: 50 µm. GCL, ganglion cell layer; INL, inner nuclear layer; IPL, inner plexiform layer; IS, inner segments; NBL, neuroblast layer; ONL, outer nuclear layer; OPL, outer plexiform layer; OS, outer segments; RPE, retinal pigment epithelium.

We previously showed that retinal cells in *Prom1*-KO mice appear to develop normally before the onset of degeneration (Dellett et al., 2015). Here, we first examined the spatiotemporal expression of *Prom1* in the mouse retina. As our *Prom1*-KO mice harbour the *lacZ* gene at the *Prom1* locus, we performed staining for β-galactosidase (β-gal) activity in the heterozygous mutant mice at postnatal day (P)2 (Fig. 1A-A″), P14 (Fig. 1B-B″), P21 (Fig. 1C-C″) and P42 (Fig. 1D-D″) in order to identify Prom1-expressing cells. At all the stages analysed, β-gal staining was localized predominantly to the outer layers – outer segments (OS), inner segments (IS) and ONL – of the retina (white brackets in Fig. 1A,A″,B,B″,C,C″,D,D″), with more sporadic staining also apparent in the inner nuclear layer (INL; arrowheads in Fig. 1B,B″,C,C″,D,D″). Given that retinal phenotypes of *Prom1*-KO mice are not obvious until 2 weeks after birth (Dellett et al., 2015), these results suggested that *Prom1* expression precedes the onset of function of the encoded protein and persists until adulthood.

### Apoptosis and an increased number of GFAP-positive glial cells observed at 3 weeks after birth in *Prom1*-KO mouse retina

**Fig. 2.**
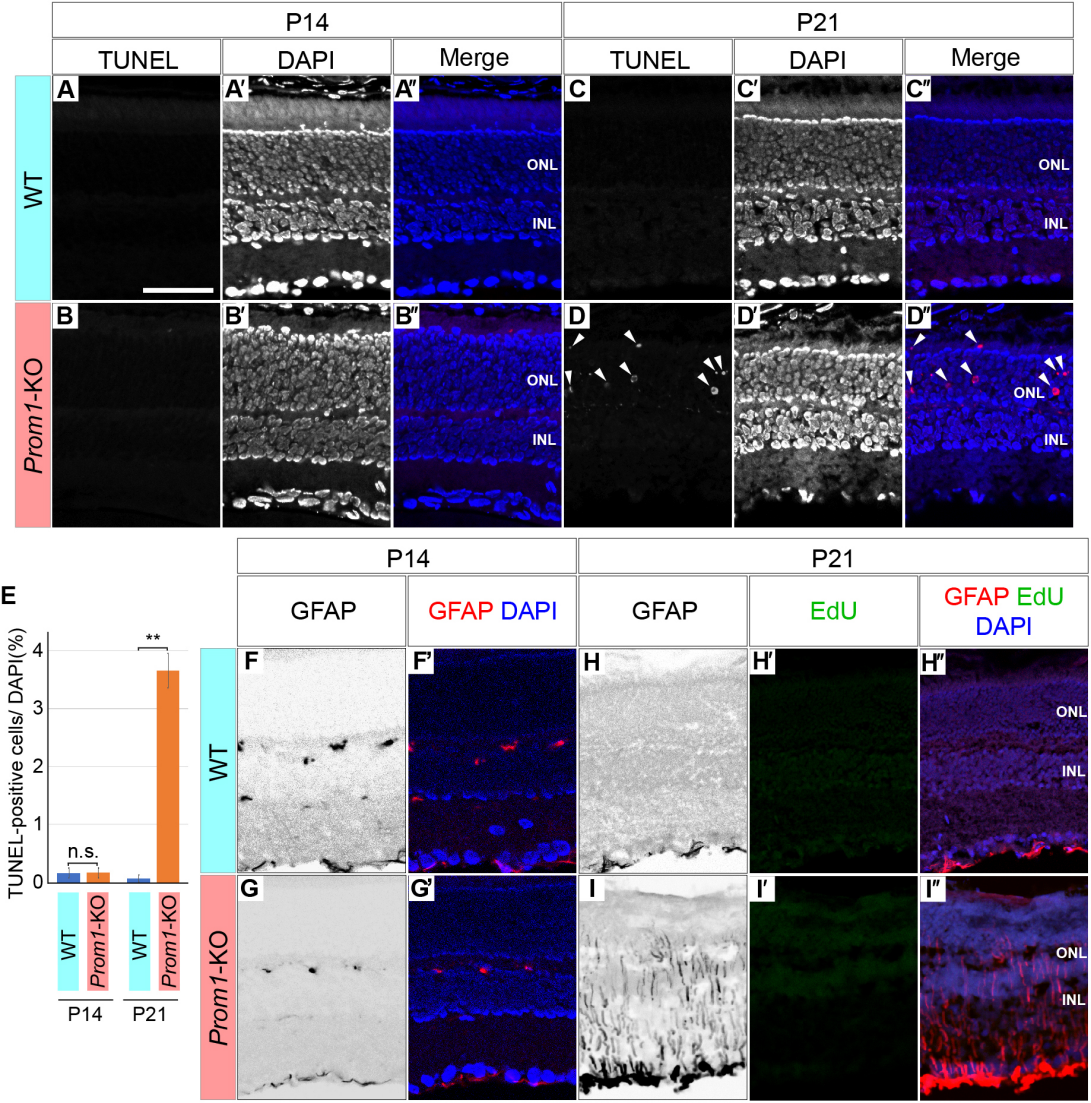
**Programmed cell death and activation of glial cells in the postnatal *Prom1*-KO mouse retina.** (A-D″) TUNEL staining of the WT (A-A″,C-C″) and *Prom1*-KO (B-B″,D-D″) mouse retina at P14 (A-B″) and P21 (C-D″). Nuclei were stained with DAPI (A′,B′,C′,D′). Merged images of TUNEL (red) and DAPI (blue) staining are also shown (A″,B″,C″,D″). Arrowheads in D and D″ indicate apoptotic cells. (E) Quantitation of the proportion of TUNEL-positive cells among all DAPI-stained cells for images similar to those in A,B,C,D. Data are means±s.e.m. for four retinas for each condition. **P<0.01; n.s., not significant (two-tailed paired Student's *t*-test). (F-I″) Immunofluorescence staining for GFAP (F,F′,G,G′,H,H′,I,I′) in the retina of WT (F-F″,H-H″) and *Prom1*-KO (G-G″,I-I″) mice at P14 (F-G′) and P21 (H-I″). The P21 retinas were also stained for incorporated EdU (H′,H″,I′,I″; see also Fig. S1). Merged images with DAPI staining are also shown (F′,G′,H″,I″). Data are representative of three (P14) or five (P21) retinas for each genotype. Scale bar: 50 µm.

We next attempted to capture the primary events at the onset of retinal degeneration in *Prom1*-KO mice. We previously showed that the *Prom1*-KO retina appears normal at P14 and begins to degenerate soon after the animals first open their eyes at this stage (Dellett et al., 2015). We therefore tested for apoptosis in the Prom1-deficient retina with the use of the terminal deoxynucleotidyl transferase-mediated dUTP nick-end labelling (TUNEL) assay to detect fragmented genomic DNA. Whereas few TUNEL-positive cells were detected in the retina of wild-type (WT) or *Prom1*-KO mice at P14 (Fig. 2A,B), a significant increase in the number of TUNEL-positive cells, located mainly in the ONL, was detected in the *Prom1*-KO retina at P21 (Fig. 2C-E). These results suggested that programmed cell death by apoptosis begins to occur in the ONL of the retina between 14 and 21 days after birth in *Prom1*-KO mice.

In addition to apoptotic cells, the appearance of glial fibrillary acidic protein (GFAP)-positive glial cells is also characteristic of RP (Rattner and Nathans, 2005; Roesch et al., 2012). GFAP is an intermediate filament protein expressed by Müller glia in response to retinal injury (Chang et al., 2007; Lewis and Fisher, 2003). We therefore examined GFAP expression in the *Prom1*-KO retina. Immunofluorescence analysis revealed that, whereas GFAP expression was apparent only sporadically in the ganglion cell layer (GCL) of the WT or *Prom1*-KO retina at P14 (Fig. 2F-G′), a marked increase in the extent of staining for GFAP was observed in the *Prom1*-KO retina, but not in the WT retina, at P21 (Fig. 2H,H″,I,I″). Assay of 5-ethynyl-2′-deoxyuridine (EdU) incorporation was performed, together with GFAP staining, in the P21 retina in order to identify cells in S phase of the cell cycle and to determine whether the increase in the number of GFAP-positive cells associated with Prom1 deficiency might be due to cell proliferation. EdU signals were not detected in the retina of WT (Fig. 2H′) or *Prom1*-KO (Fig. 2I′) mice, whereas EdU was incorporated into cells of the cornea of the same animals (Fig. S1), suggesting that EdU was available in the eye and that the increase in the number of GFAP-positive cells in the *Prom1*-KO retina at P21 was due to a change in the nature of glial cells already present in the retina rather than to cell proliferation.

Together, these findings indicated that retinal development remains intact in *Prom1*-KO mice until P14, after which an increase in the numbers of GFAP-positive glial cells and apoptotic photoreceptors occurs in association with eye opening and the onset of retinal degeneration.

### Altered gene expression in the *Prom1*-KO mouse retina

**Fig. 3.**
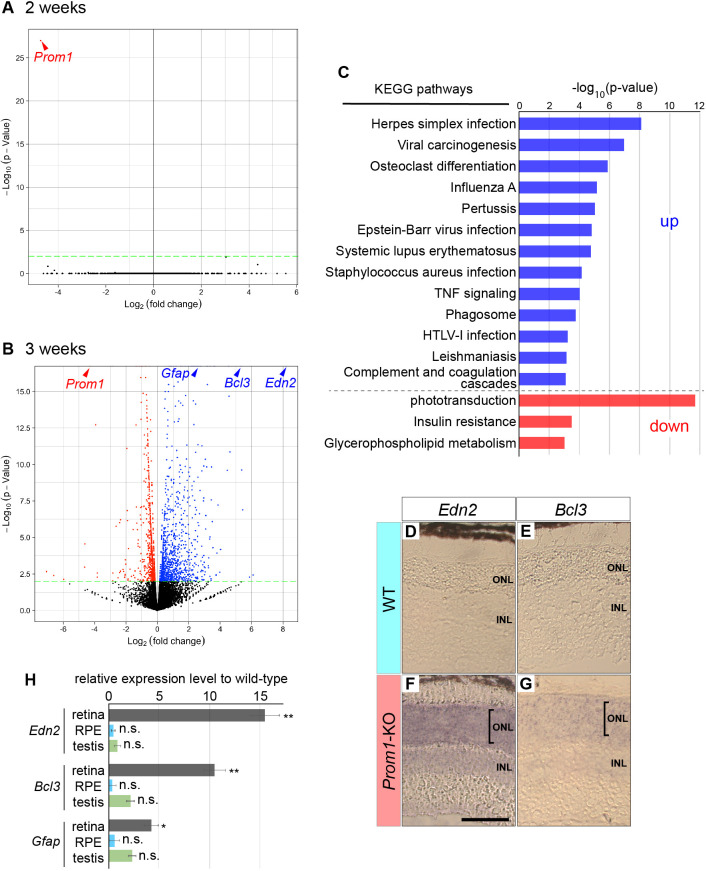
**Effects of Prom1 deficiency on gene expression in the retina.** (A,B) Volcano plots for RNA-sequencing analysis of the retina of *Prom1*-KO mice relative to that of WT mice at P14 (A) and P21 (B). Genes with a *P*-value <1×10^–17^, including *Edn2*, *Bcl3*, *Gfap* and *Prom1*, are shown on the ceiling line in B. Genes upregulated are indicated by blue dots and those downregulated are indicated by red dots. A cut-off *P*-value of 1×10^–2^ is indicated by the green dashed line. Data are for three (P14) or four (P21) retinas of each genotype. (C) Pathways either upregulated (up) or downregulated (down) with a *P*-value <1×10^−2^ in the *Prom1*-KO retina based on Kyoto Encyclopedia of Genes and Genomes (KEGG) analysis of the RNA-sequencing data at P21. (D-G) *In situ* hybridization analysis of *Edn2* (D,F) and *Bcl3* (E,G) expression in the retina of WT (D,E) and *Prom1*-KO (F,G) mice at P21. Scale bar: 50 µm. Predominant expression in the ONL is indicated by the brackets in F and G. (H) RT-qPCR analysis of *Edn2*, *Bcl3* and *Gfap* expression in the retina, RPE and testis of *Prom1*-KO mice relative to WT mice at P21. Data are means±s.e.m. for three specimens of each genotype. *P<0.05, **P<0.01; n.s., not significant (two-tailed paired Student's *t*-test).

Our results suggested that a critical point for retinal degeneration in *Prom1*-KO mice exists between P14 and P21. We therefore next sought to identify genes for which expression might be affected by Prom1 deficiency by subjecting the retina of WT and *Prom1*-KO mice at P14 and P21 to high-throughput expression analysis based on RNA sequencing. Whereas variations in gene expression were apparent within each genotype at P14, the only gene for which expression differed significantly between genotypes was *Prom1* itself (Fig. 3A; Table S1), suggesting that Prom1 does not significantly influence the gene expression profile at this stage. In contrast, the expression of various genes differed between the two genotypes at P21 (Fig. 3B; Table S2). The expression of 1081 (indicated by blue dots; Fig. 3B) and 765 genes (indicated by red dots; Fig. 3B) was up- and downregulated, respectively, in the *Prom1*-KO retina, with a *P*-value of <0.01. In particular, expression of *Edn2* was the most consistently and markedly upregulated in the *Prom1*-KO retina. In addition, consistent with the associated increase in the numbers of apoptotic and GFAP-positive cells (Fig. 2I), expression of the apoptosis-related gene *Bcl3* and the glial marker *Gfap* was increased in the *Prom1*-KO retina at P21. Conversely, the expression of genes related to RP or of those essential for retinal development and functional homeostasis – including *Fscn2* (RP30) (Wada et al., 2001), *Prph2* (RP7) (Conley and Naash, 2014), *Nr2e3* (RP37) (Cheng et al., 2004), *Kcnv2* (Hölter et al., 2012), *Elovl2* (Chen et al., 2020), *Pde6b* (RD1) (Yeo et al., 2019) and *Ttc21b* (Liu et al., 2010) – was downregulated in the *Prom1*-KO retina at P21 (Table S2). Moreover, gene ontology (GO) analysis revealed that pathways related to responses to infection or to phototransduction were up- and downregulated, respectively, in the *Prom1*-KO retina at P21 (Fig. 3C; Table S3), suggesting that Prom1 plays key roles in retinal homeostasis.

We next attempted to localize the areas of *Edn2* and *Bcl3* expression in the P21 retina by *in situ* hybridization. Whereas signals for both of these genes were essentially absent from the WT retina (Fig. 3D,E), they were detected predominantly in the ONL of the *Prom1*-KO retina (Fig. 3F,G), with a low level of *Edn2* expression also being detected in the INL, consistent with previous findings (Bramall et al., 2013). These results suggested that the expression of *Edn2* and *Bcl3* is induced mainly in photoreceptor cells of the mutant retina.

We further investigated whether the observed effects of Prom1 deficiency on gene expression were specific to the retina. As *Prom1* is expressed in the retina, retinal pigment epithelium (RPE) and testis (Fargeas et al., 2004), we performed reverse transcription (RT) and quantitative polymerase chain reaction (qPCR) analysis of RNA prepared from these tissues of WT and *Prom1*-KO mice at P21. Consistent with the results of our RNA-sequencing analysis, the expression of *Edn2*, *Bcl3* and *Gfap* was increased in the retina of *Prom1*-KO mice (Fig. 3D). However, the expression of these genes in the RPE and testis did not differ between the two genotypes, indicating that the effect of Prom1 on their expression is specific to the retina.

Together, our expression analyses indicated that Prom1 deficiency results in upregulation of infectious response-related genes and downregulation of genes essential for functional homeostasis of photoreceptor cells of the *Prom1*-KO retina at 3 weeks after birth. In addition, the alteration of the gene expression occurs in various cell types.

### Gliogenesis and cell death-related gene expression induced by light stimulation in the *Prom1*-KO mouse retina

**Fig. 4.**
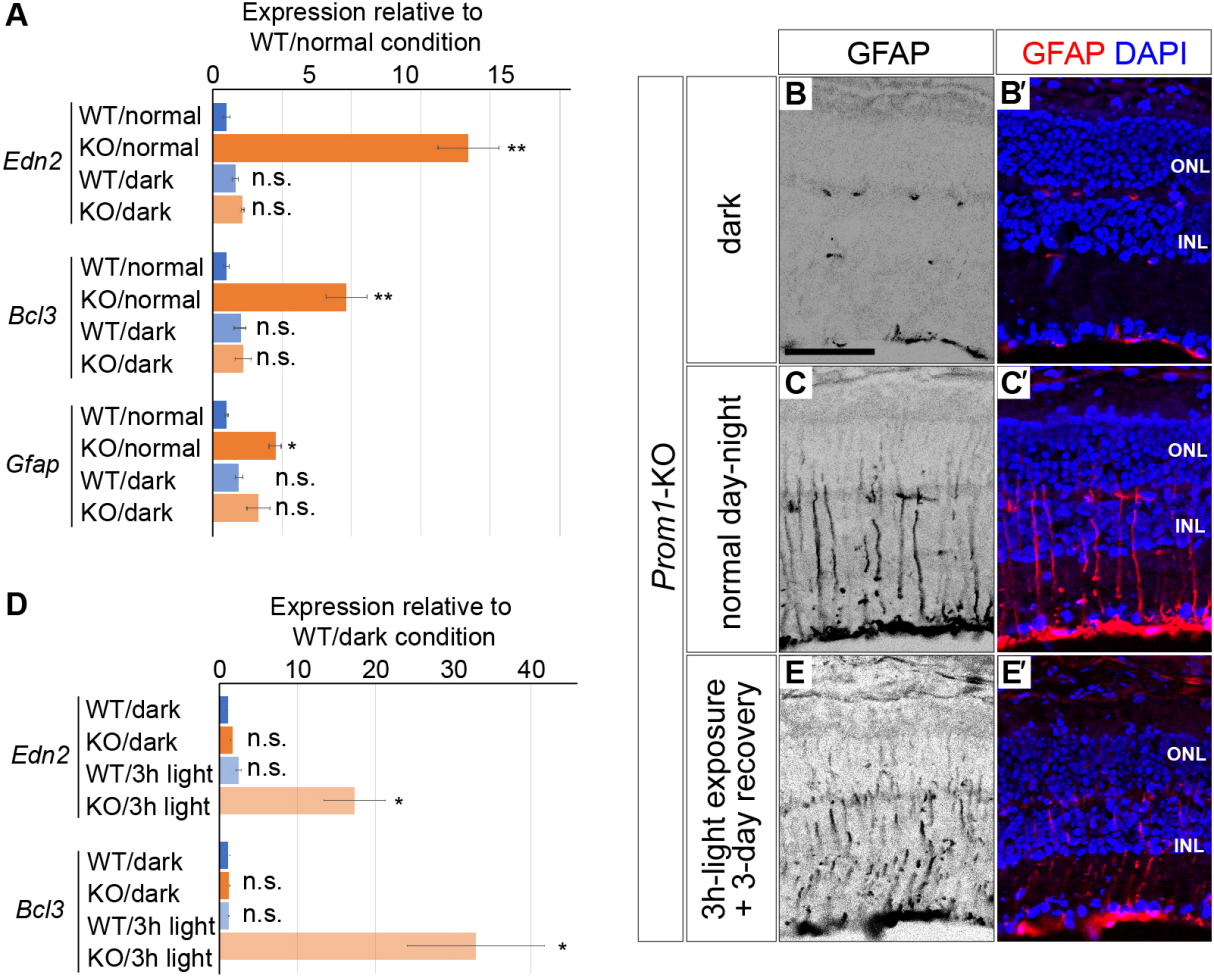
**Genes with expression increased by Prom1 deficiency are upregulated by light stimulation.** (A) RT-qPCR analysis of *Edn2*, *Bcl3* and *Gfap* expression in the P21 retina of WT or *Prom1*-KO mice that had been reared under a normal day-night cycle or in the dark. Data are means±s.e.m. for four retinas for each condition. *P<0.05, **P<0.01, n.s. (not significant) versus WT/normal (one-way ANOVA followed by Tukey's post hoc test). (B-C′) Immunofluorescence analysis of GFAP expression in the retina of *Prom1*-KO mice raised as in A (B,C). Merged images with DAPI staining are also shown (B′,C′). Scale bar: 50 µm. Data are representative of four (dark) or seven (normal day-night) retinas. (D) RT-qPCR analysis of *Edn2* and *Bcl3* expression in the retina of *Prom1*-KO and WT mice that had been reared in the dark condition for 3 weeks and then exposed (or not) to a bright light for 3 h. Data are means±s.e.m. for five retinas for each condition. *P<0.05, n.s. versus WT/dark (one-way ANOVA followed by Tukey's post hoc test). (E,E′) Immunofluorescence analysis of GFAP expression in the retina of *Prom1*-KO mice raised in the dark and stimulated with light as in D and then allowed to recover in the dark for 3 days (E). Merged image with DAPI staining is also shown (E′). Data are representative of three retinas.

Given that mice open their eyes around P14, we reasoned that the aberrant gene expression in the *Prom1*-KO retina at P21 might be triggered by light stimulation. To address this hypothesis, we reared *Prom1*-KO mice either under a normal day-night cycle or in the dark until P21 and then subjected the retina to RT-qPCR analysis of gene expression. Whereas the expression of *Edn2*, *Bcl3* and *Gfap* did not differ between *Prom1*-KO and WT mice reared in the dark condition, marked upregulation of the expression of each of these genes was apparent specifically in *Prom1*-KO mice raised under the normal day-night condition (Fig. 4A). Consistent with these results, immunofluorescence analysis showed that the number of GFAP-positive cells in the retina was smaller for *Prom1*-KO mice reared in the dark compared with those reared under the normal condition (Fig. 4B,C). These results indicated that light stimulation is a major cause of the aberrant gene expression apparent in the *Prom1*-KO retina at P21.

To examine further the effect of light on gene expression, we maintained *Prom1*-KO mice and their WT littermates under the dark condition for 3 weeks and then exposed them to a bright light for 3 h. RT-qPCR analysis revealed that light stimulation resulted in a marked increase in the expression of both *Edn2* and *Bcl3* in the retina of *Prom1*-KO mice but not in that of WT mice (Fig. 4D), suggesting that induction of the expression of these genes is a primary response to light stimulation. In addition, allowing the mice to recover for 3 days in the dark after the 3-h light stimulation revealed an increase in the number of GFAP-positive cells in the retina of *Prom1*-KO mice (Fig. 4E). Collectively, these results suggested that the upregulation of *Edn2*, *Bcl3* and *Gfap* expression apparent in the retina of *Prom1*-KO mice is an immediate response to light stimulation and is followed by the increase in the number of GFAP-positive glial cells detected by immunofluorescence analysis.

### Endothelin receptor antagonists attenuate photoreceptor death and *Gfap* expression in the *Prom1*-KO mouse retina

To establish a drug-based therapeutic approach for the retinal pathology of *Prom1*-KO mice, we focused on endothelin signalling, as the expression of *Edn2* was most prominently induced in the mutant retina at the early stage of degeneration. Endothelin acts at specific receptors (Patel et al., 2014; Sarthy et al., 2015) to induce retinal cell death (Kobayashi et al., 2005) and to increase the number of GFAP-positive Müller glial cells (Rattner and Nathans, 2005; Rattner et al., 2008; Yuen et al., 2013). Given the elevated expression of *Edn2* and *Gfap* apparent in the retina of *Prom1*-KO mice, we hypothesized that ET-2, the mature form of the *Edn2* product (Goldman, 2014), might induce the GFAP expression apparent in association with retinal degeneration in the Prom1-deficient animals. We therefore examined the possible effects of endothelin receptor antagonists in *Prom1*-KO mice.

**Fig. 5.**
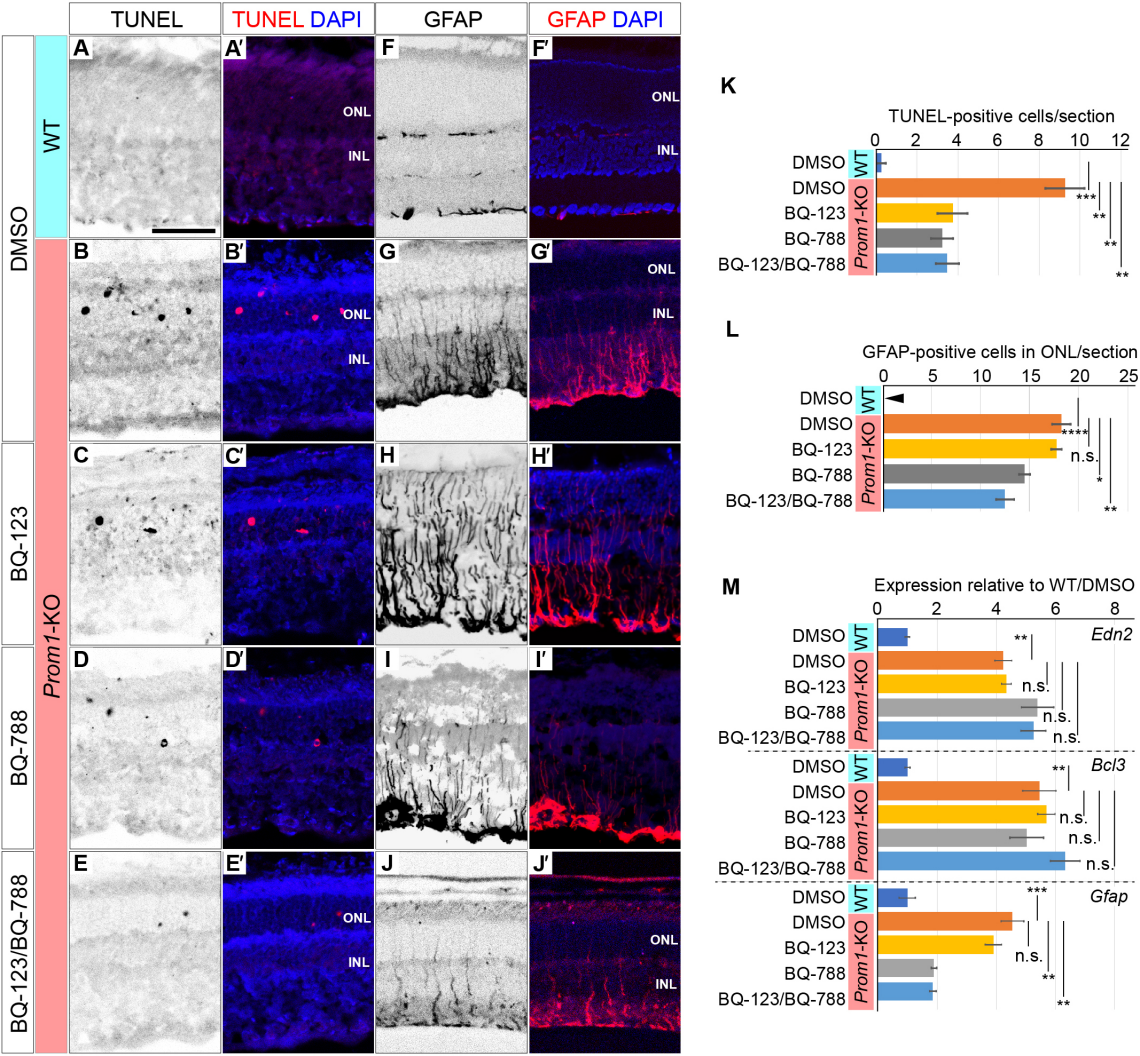
**Endothelin receptor antagonists reduce the numbers of TUNEL-positive cells and GFAP-positive glial cells in the *Prom1*-KO retina.** (A-J′) TUNEL staining for apoptotic cells (A-E′) and immunofluorescence analysis of GFAP expression (F-J′) in the retina of WT (A,A′,F,F′) or *Prom1*-KO (B-E′,G-J′) mice treated with DMSO (A′,B′,F′,G′), BQ-123 (C,C′,H,H′), BQ-788 (D,D′,I,I′), or the combination of BQ-123 and BQ-788 (E,E′,J,J′) at P14, P19 and P24 and analysed at P28. Merged images with DAPI staining are also shown (A′,B′,C′,D′,E′,F′,G′,H′,I′,J′). Scale bar: 50 µm. (K) Number of apoptotic cells per section determined from images as in A,B,C,D,E. (L) Number of GFAP-positive cells in the ONL per section determined from images as in F,G,H,I,J. The arrowhead indicates zero. (M) RT-qPCR analysis of *Edn2*, *Bcl3* and *Gfap* expression in the retina of the treated mice. All quantitative data in K-M are means±s.e.m. for four retinas per condition. *P<0.05, **P<0.01, ***P<0.001; n.s., not significant (one-way ANOVA followed by Tukey's post hoc test).

BQ-123 and BQ-788 are antagonists of endothelin receptors A (EdnrA) and B (EdnrB), respectively (Fukuroda et al., 1994), both of which mediate the actions of ET-2 (Bramall et al., 2013; Rosanò et al., 2013). WT or *Prom1*-KO mice were injected intraperitoneally with dimethyl sulfoxide (DMSO) vehicle, BQ-123, BQ-788 or both drugs at P14, P19 and P24, and the mice were analysed at P28. Whereas the numbers of TUNEL-positive and GFAP-positive cells were increased in the retina of *Prom1*-KO mice treated with DMSO compared with WT control animals (Fig. 5A-B′,F-G′), the number of TUNEL-positive cells was significantly reduced by treatment of the mutant mice with BQ-123, BQ-788 or both drugs (Fig. 5B-E′,K), suggesting that both antagonists reached the retina and attenuated photoreceptor degeneration. In contrast, the number of GFAP-positive cells in the mutant retina was reduced by treatment with BQ-788 but not by that with BQ-123 (Fig. 5G-J′,L), suggesting that BQ-123 and BQ-788 have differential effects on Müller glial cells.

We also examined the effects of BQ-123 and BQ-788 on retinal expression of *Edn2*, *Bcl3* and *Gfap* by RT-qPCR analysis (Fig. 5M). Expression of each gene at P28 was increased in the retina of DMSO-treated *Prom1*-KO mice compared with that of DMSO-treated WT mice. Whereas neither BQ-123 nor BQ-788 affected the expression of *Edn2* and *Bcl3* in the mutant retina, *Gfap* expression was significantly inhibited by BQ-788 but not by BQ-123. These findings thus suggested that inhibition of signalling by either EdnrA or EdnrB improves photoreceptor survival, whereas that of signalling by EdnrB attenuates GFAP-positive Müller glial cells.

### Effects of endothelin receptor antagonists on retinal vascular narrowing in the *Prom1*-KO retina

**Fig. 6.**
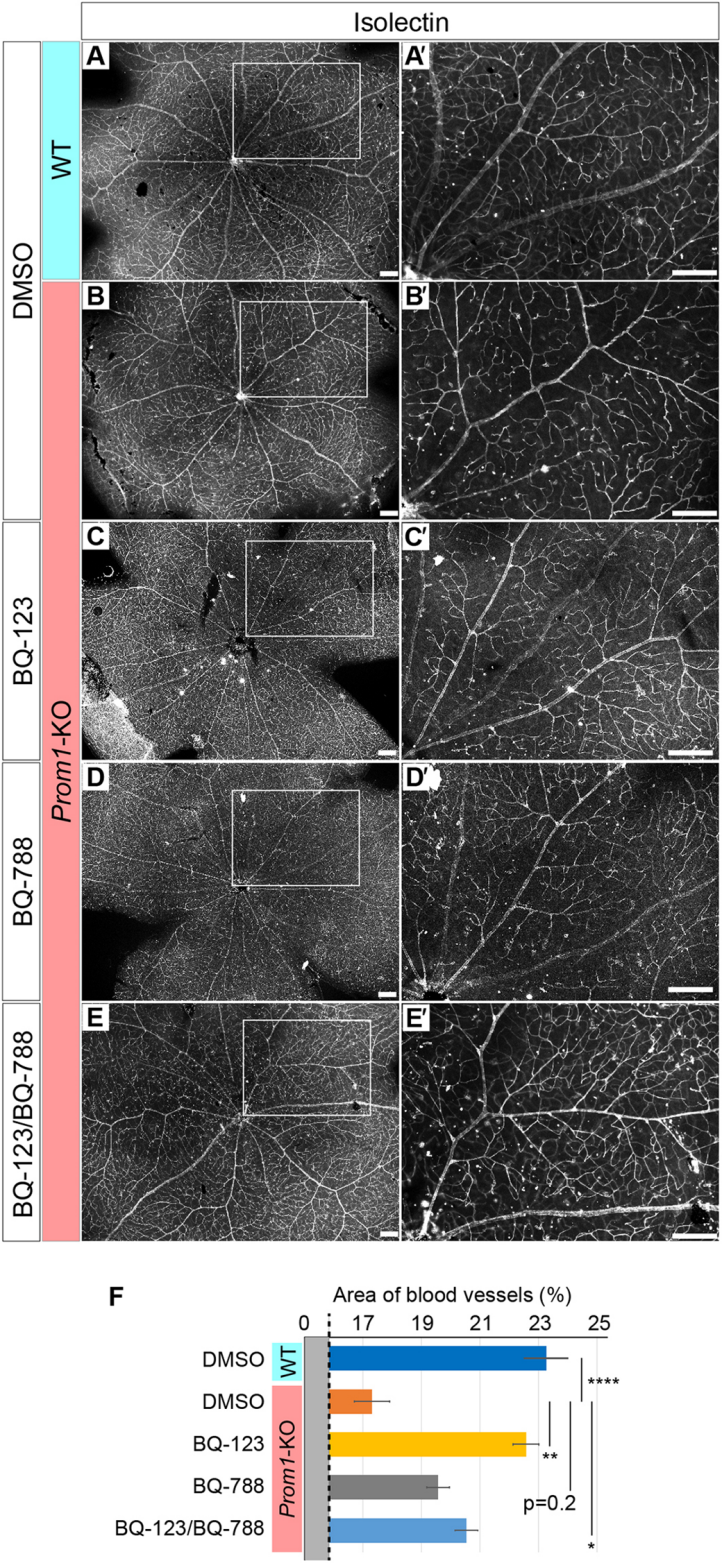
**Endothelin receptor antagonists attenuate vascular stenosis in the retina of *Prom1*-KO mice.** (A-E′) Isolectin staining of the retina of mice treated with BQ-123 and BQ-788 as in Fig. 5 (A,B,C,D,E). The boxed regions of the left panels are shown at higher magnification in A′,B′,C′,D′,E′. Scale bars: 100 µm. (F) Area of blood vessels measured in images similar to those in A,B,C,D,E. Data are means±s.e.m. for five retinas per condition. *P<0.05, **P<0.01, ****P<0.0001 (one-way ANOVA followed by Tukey's post hoc test).

Retinal vascular narrowing, or stenosis, has previously been identified in the Prom1-deficient mouse retina (Zacchigna et al., 2009) and is a common feature of individuals with RP (Ma et al., 2012). We therefore also examined the possible effects of the endothelin receptor antagonists BQ-123 and BQ-788 on retinal vessel stenosis in *Prom1*-KO mice. We visualized retinal vascular endothelial cells by staining of flat-mount preparations of the retina with fluorescently labelled isolectin (Weerasekera et al., 2015). We confirmed that retinal vessels were intact in WT mice injected with BQ-123, BQ-788 or both drugs (Fig. S2). Large vessels appeared narrower and their density was reduced in the retina of DMSO-treated mutant mice at P28 compared with that of DMSO-treated WT mice (Fig. 6A,A′,B,B′,F), which was consistent with the previous findings (Zacchigna et al., 2009). However, the diameter and density of the retinal vessels were largely normalized by treatment of the mutant animals with BQ-123 (Fig. 6C,C′,F). BQ-788 also tended to improve the density of the vessels; however, the effect was significantly weaker than that of BQ-123 (Fig. 6D,D′,F). The double treatment with BQ-123 and BQ-788 also recovered the vessel constrictions, although the extent was comparable with that with the individual injections (Fig. 6E,E′,F).

Together, the findings suggest that the activation of EdnrA signalling contributes to the vascular stenosis characteristic of the *Prom1*-KO mouse retina, and that BQ-123 is predominantly influential to these vascular cells.

## DISCUSSION

### The retina develops normally but undergoes light-dependent changes in gene expression in *Prom1*-KO mice

We have here described early manifestations of the retinal degeneration that occurs in *Prom1*-KO mice and identified genes related to this process. We thus detected the aberrant presence of GFAP-positive glial cells and the expression of genes associated with responses to infection in the mutant retina. Given that the expression of these genes was not activated in the retina of *Prom1*-KO mice maintained in the dark condition, it appears to be dependent on light stimulation. Finally, we found that the deterioration and gliosis characteristics of the mutant retina were ameliorated by the administration of endothelin receptor antagonists.

Although we found that *Prom1* is expressed in the retina from birth (Fig. 1A-A″), the loss of Prom1 did not substantially affect the expression level of any gene in the retina at P14, when mice first open their eyes, suggesting that Prom1 may not play an essential role in the retina prior to light exposure. We previously showed by RT-qPCR analysis that the expression of both *Rdh12* and *Abca4*, two genes that contribute to the visual cycle, was reduced in the retina of *Prom1*-KO mice compared with that of WT mice at P14 (Dellett et al., 2015), and we suggested that impairment of the visual cycle might lead to retinal degeneration. Although the decrease in the expression level of these genes is reproducible as assayed by RT-qPCR analysis (Fig. S3), the difference in the expression of each gene between the two genotypes was associated with a relatively high *P*-value in the high-throughput expression analysis performed in the present study (Fig. 3; Table S1), suggesting that this alteration does not primarily attribute to the photoreceptor degeneration.

In contrast to the lack of critical changes at P14, we detected a number of genes with altered expression in the *Prom1*-KO retina at P21. In particular, the expression of genes related to phototransduction or to RP was significantly downregulated in the *Prom1*-KO retina at P21, indicating that Prom1 may be an essential regulator of the transcription of these genes or may form a transcriptional network with them. Consistent with this finding, the phenotypes found in knockout mice of these downregulated genes are similar to those of *Prom1*-KO mice (Ferrari et al., 2011) and include night blindness and progressive loss of vision.

In addition to the photoreceptor-related genes, the expression of genes related to insulin resistance and metabolism was also downregulated in the *Prom1*-KO retina at P21 (Fig. 3C; Table S2). These genes are also related to vascular function (Järgen et al., 2017), consistent with the vascular stenosis characteristic of the *Prom1*-KO retina (Fig. 6) (Zacchigna et al., 2009).

In contrast to the downregulated genes, the genes related to responses to infection, including those for proteins that play a role in interferon or tumour necrosis factor (TNF)-related signalling, was upregulated in the mutant retina at P21. Given that such signalling has also been implicated in programmed cell death of neurons (Probert, 2015), this finding is consistent with our detection of apoptotic cells in the retina of *Prom1*-KO mice at the onset of retinal degeneration (Fig. 2D-D″).

Of the genes with upregulated expression in the *Prom1*-KO retina, *Edn2* showed the largest fold change. The expression of *Edn2* has also been shown to be upregulated in other mouse models of RP (Bramall et al., 2013), including retina-specific *Cdhr1*-KO mice (Rattner and Nathans, 2005), with Prom1 and Cdhr1 having been found to interact with each other (Yang et al., 2008). Although a recent study demonstrated different phenotypes for the *Prom1-* and *Cdhr1*-mutant retinas (Carr et al., 2021), the functions of these two proteins may be mediated by similar signalling pathways.

Although we found that the expression of *Edn2* and *Bcl3* in the *Prom1*-KO retina was rapidly induced by light stimulation, the mechanisms underlying this effect remain unclear. Nevertheless, given that we previously showed that Prom1 regulates chloride conductance activated by intracellular calcium uptake (Hori et al., 2019), an imbalance in intracellular ions triggered by the loss of Prom1 may impair the function of cytoplasmic organelles such as mitochondria and the endoplasmic reticulum, and thereby elicit a stress response. Studies to identify the transcriptional regulatory elements of *Edn2* and *Bcl3* and the corresponding transcription factors and upstream signalling pathways underlying their photoactivation are warranted.

In the present study, whole-retinal tissue was subjected to transcriptome analysis, with the identified changes in gene expression likely occurring in different cell types. The upregulated expression of GFAP in the mutant retina was thus detected in Müller glial cells (Fig. 2H,H″,I,I″), whereas that of *Edn2* and *Bcl3* was apparent in the ONL (Fig. 3D-G), where photoreceptor cells reside, and many of the other affected genes are expressed in different cell types (Rattner et al., 2008). It is thus difficult to identify the cell types in which changes in gene expression occur by such transcriptome analysis of bulk tissue, and the possibility that observed expression changes take place in only a small population of cells cannot be excluded. More detailed analysis, such as by single-cell RNA sequencing, should allow alterations in the expression of specific genes to be mapped to specific cells.

### Blocking of endothelin signalling ameliorates retinal phenotypes of Prom1 deficiency

Gliosis, a phenotype we identified in *Prom1*-KO mice, is a common feature of RP (de Hoz et al., 2016; Massengill et al., 2020; Roche et al., 2018). Gliosis is characterized by the upregulation of *Gfap* expression and also occurs in various other neurodegenerative conditions, including in association with central nervous system damage and the recovery process (Burda and Sofroniew, 2014; Sardar Pasha et al., 2017; Sarthy et al., 2015). GFAP-positive glial cells mediate the phagocytosis of dead photoreceptor cells (Sakami et al., 2019), and the glial cells that we detected extending into the ONL of the *Prom1*-KO retina at P21 (Fig. 2I) may therefore function to remove dead cells and to maintain retinal homeostasis (Sarthy et al., 2015).

A relation between *Edn2*/ET-2 and gliosis has been suggested by previous studies (Burda and Sofroniew, 2014; Gadea et al., 2008; Rattner and Nathans, 2005; Swiderski et al., 2007), and we focused on the potential roles of ET-2 signalling in photoreceptor apoptosis (Fig. 5) and retinal vessel stenosis (Fig. 6) in the *Prom1*-KO retina. *Edn2* produces the secretory peptide ET-2 that plays a role in a wide range of biological processes, including smooth muscle contraction and ovulation (Cacioppo et al., 2014), as well as development of the enteric nervous system (Gershon, 1999). Its expression is also induced in association with the inflammatory response and promotes glial cell activation in the central nervous system (Yuen et al., 2013). The vascular narrowing detected in the retina of Prom1-deficient mice in both the present and previous (Zacchigna et al., 2009) studies is consistent with the constriction of retinal venules observed in response to treatment with endothelin (Chen et al., 2018). Furthermore, the administration of ET-2 in the retina was previously shown to induce Müller cell damage and the infiltration of macrophages (Alrashdi et al., 2018), and loss of function of *Edn2* was found to increase photoreceptor survival (Bramall et al., 2013). Aberrant upregulation of *Edn2*/ET-2 therefore appears to have adverse effects on retinal homeostasis. On the other hand, overexpression of ET-2 in RP model mice suggested that ET-2 also promotes photoreceptor cell survival (Bramall et al., 2013). These various observations suggest that a basal level of *Edn2*/ET-2 expression is essential for homeostasis in the retina, but that marked changes in such expression can have negative effects.

In our study, we examined the effects of BQ-123 and BQ-788, two endothelin receptor antagonists adopted in previous studies (Fukuroda et al., 1994), on *Prom1*-KO retinal phenotypes. We found that BQ-788 reduced the number of GFAP-positive glial cells (Fig. 5I,I′,L), whereas BQ-123 promoted the recovery of retinal vessels (Fig. 6C,C′,F), in *Prom1*-KO mice. These differential effects likely reflect the differential expression of EdnrA and EdnrB in the retina. Both EdnrA and EdnrB are expressed in choroidal and retinal vessels (Bramall et al., 2013; Stitt et al., 1996), and EdnrB is expressed in Müller and horizontal cells (Alrashdi et al., 2018; Bramall et al., 2013; Rattner et al., 2013). BQ-788 may therefore block EdnrB signalling in Müller cells (Fig. 5I-I′,L), whereas BQ-123 may block EdnrA signalling in retinal vascular cells (Fig. 6C,C′,F). We found that the administration of both BQ-123 and BQ-788 ameliorated all examined phenotypes, including photoreceptor apoptosis (Fig. 5E,E′,K), upregulation of GFAP expression (Fig. 5J,J′,L) and vascular narrowing (Fig. 6E,E′,F), in the retina of *Prom1*-KO mice. EdnrA and EdnrB are expressed at only low levels, if at all, in photoreceptor cells (Bramall et al., 2013). However, the number of apoptotic cells in the mutant retina was reduced by administration of either BQ-123 or BQ-788. This effect of the antagonists is therefore likely indirect, and may be a secondary consequence of improvement of the retinal vasculature or of other actions such as induction of neurotrophic factors.

Choroidal vessels, located below the RPE (Zheng et al., 2016), which bring oxygen and nutrients to RPE and ONL, were not substantially affected in *Prom1*-KO mice, at least up to 4 weeks of age (Fig. S4), suggesting that these vessels are less sensitive to endothelin signalling than are retinal vessels. Studies on the direct effects of endothelins on various cell types in the retina would be facilitated by analysis of *EdnrA* and *EdnrB* mutant mice. In addition, as gliosis is a common feature of RP, it will be important to test whether the endothelin receptor antagonists tested in this study might ameliorate the phenotypes of other models of this disease.

Caution is warranted, however, with regard to blockade of endothelin signalling as a potential clinical strategy for the treatment of RP, given the various functions of endothelins and the possible adverse effects of long-term administration of endothelin receptor antagonists at the systemic level (Cacioppo et al., 2014; Kedzierski and Yanagisawa, 2001). In the present study, we administered the antagonists for only 2 weeks in mice at a young age. Topical administration such as by intravitreal injection may help to minimize possible side effects of such drugs. Gene therapy, such as that targeting endothelin signalling, is also a potential therapeutic approach for RP (Cehajic-Kapetanovic et al., 2020).

Characterization of the early key steps of retinal degeneration may inform the development of new treatments that preserve photoreceptor cells via modulation of signalling that is altered in affected individuals. Pharmacological treatment of individuals at risk for the development of RP or at the early phases of its manifestation may thus delay the onset or slow the progression of disease. Whereas several agents have been proposed as therapeutic agents for RP, none has proved to be generally effective. Our present results may provide a basis for the establishment of new therapeutic strategies for this and other incurable retinal diseases.

## MATERIALS AND METHODS

### Mice and their treatment

All animal experiments were approved by the animal welfare and ethics committees of both Yamaguchi University (approval numbers J16021 and U16005 for K.K.) and Nara Institute of Science and Technology (approval numbers 1810, 311, and 389 for N.S.) and were performed in accordance with the relevant guidelines and regulations. *Prom1*-KO mice were established previously (CDB0623K, http://www2.clst.riken.jp/arg/methods.html). As a result of difficulty with their breeding on the pure C57BL/6 background (Dellett et al., 2015), *Prom1*-KO mice were reared on a hybrid genetic background of C57BL/6 and CBA/NSlc strains. The targeting vector for *Prom1* ablation contained the *lacZ* (β-gal) gene, with the result that expression of this latter gene reflects that of *Prom1*. Both the *Prom1*-KO mice and their WT littermates were kept on a 12-h-light, 12-h-dark cycle, with the cage racks being covered with blackout curtains and all procedures including feeding and cage maintenance being performed in the absence of light (<0.5 lux) during the dark phase. For assay of EdU incorporation, mice were killed by cervical dislocation at 2 h after intraperitoneal injection of EdU (Thermo Fisher Scientific) at 60 mg/kg, and eyes were removed for retinal staining with an EdU-staining proliferation kit (ab219801, Abcam). For experiments involving light stimulation, mice were exposed for 3 h to a light panel (LED viewer 5000; Shinko, Tokyo, Japan) placed on top of the cage, which resulted in a light intensity of 3800 lux at the bottom of the cage. For chemical treatment, mice received intraperitoneal injections (2 mg/kg) of the endothelin receptor antagonists BQ-123 (ab141005, Abcam) or BQ-788 (ab144504, Abcam) on P14, P19 and P24. The mice were then subjected to analysis at P28.

### RNA extraction and RT-qPCR analysis

The retina, RPE and testis were dissected from mice of the indicated genotypes. Total RNA was extracted from the isolated tissue and was subjected to RT with the use of a NucleoSpin RNA extraction kit (U955C, Takara) and PrimeScript RT reagent kit (RR037, Takara), respectively. The resulting cDNA was subjected to qPCR analysis with a CFX qPCR machine (Bio-Rad) and with the primers listed in Table S4. The amplification data were analysed with the comparative *C*_t_ method, and gene expression levels were normalized by that of the glyceraldehyde-3-phosphate dehydrogenase gene (*Gapdh*).

### High-throughput expression analysis

Total RNA samples were prepared from three (P14) or four (P21) retinas of WT or *Prom1*-KO mice and were used to synthesize cDNA libraries with a TruSeq stranded-mRNA library preparation kit (20020594, Illumina). The libraries were sequenced with the NextSeq 500 platform (Illumina). A total of ∼20 million reads per sample was mapped with the use of the CLC genomics workbench software (Qiagen) (Robinson et al., 2010). GO term analysis was performed on the DAVID website (https://david.ncifcrf.gov/tools.jsp) according to the Kyoto Encyclopedia of Genes and Genomes (KEGG) database (https://www.genome.jp/kegg).

### Immunofluorescence, β-gal, TUNEL staining and *in situ* hybridization

For immunofluorescence analysis, the enucleated retina was fixed for 2 h with a mixture of 1% paraformaldehyde and 0.2% glutaraldehyde in phosphate-buffered saline (PBS), incubated overnight in PBS containing 15% sucrose, embedded in OCT compound (Sakura) and sectioned at a thickness of 12 µm. The sections were exposed to mouse monoclonal antibodies to GFAP (G3893, Sigma-Aldrich), and immune complexes were detected with Cy3-conjugated secondary antibodies (715-166-151, Jackson ImmunoResearch). Nuclei were counterstained with 4′,6-diamidino-2-phenylindole (DAPI) with the use of DAPI Fluoromount-G (0100-20, Southern Biotech). Sections were also stained for β-gal activity with the use of a staining kit (11828673001, Roche). Apoptotic cells were detected by TUNEL analysis with digoxigenin (DIG)-labelled dUTP (S7105, Merck Millipore), terminal deoxynucleotidyl transferase (3333566001, Merck) and rhodamine-conjugated antibodies to DIG (11207750910, Roche).

For making DIG-labelled probes of *Edn2* and *Bcl3* used for *in situ* hybridization, the fragments of these genes were amplified by RT-PCR with the primers (528 bp for *Edn2*; forward, TATAGAATTCATGGTCTCCGCCTGGTGTTCCATCGCTCTG and reverse, TATACTCGAGTTATCTCTTCCTCCATCTAGAGTATGCAGG; and 800 bp for *Bcl3*; forward, TATAGAATTCTAACATAGCCGCTGTCTACCGAATACTCAG and reverse, TATACTCGAGAGCCAGGAGCATCTTTCGGGGGAGACAGCG), and were subcloned into the pBluescript-SK vector at the EcoRI and XhoI sites. The antisense probes were synthesized with the T7 RNA polymerase (P2075, Promega) and DIG RNA-labelling mix (11277073910, Sigma-Aldrich).

*In situ* hybridization was performed as described previously (Sasai et al., 2014; Yatsuzuka et al., 2019). Briefly, the hybridization was performed at 65°C with buffer containing 5× SSC [0.75 mol/l sodium chloride (NaCl), 0.075 mol/l sodium citrate; pH 5.0], 5× Denhardt's reagent (750018, Thermo Fisher Scientific), 500 µg/ml Salmon Sperm DNA (15632011, Thermo Fisher Scientific), 500 µg/ml Torula RNA (R3629, Sigma-Aldrich), 0.1 mg/ml heparin sodium (085-00134, Wako), 1 mM ethylenediaminetetraacetic acid (EDTA), 0.1% (v/v) 3-[(3-Cholamidopropyl) dimethylammonio] propanesulfonate (CHAPS) and 50% formamide. For developing the signals, anti-DIG-alkaline phosphatase (AP)-conjugated antibody (11093274910, Sigma-Aldrich) and BCIP/NBP solution (B6404, Sigma-Aldrich) were used.

### Isolectin staining

For preparation of flat-mount samples, the retina was fixed for 150 min with 4% paraformaldehyde and the RPE was peeled off. The samples were subjected to isolectin staining by consecutive exposure to 5% dried skim milk and Alexa Flour 488-conjugated GS-IB4 (I21411, Thermo Fisher Scientific) as described previously (Yamaguchi et al., 2016).

### Image acquisition and processing

All images were acquired with a BZ-X710 microscope (Keyence) or an LSM 710 confocal microscope (Zeiss), and imaging data were processed and integrated with Photoshop (Adobe) and Illustrator (Adobe) software.

### Statistical analysis

Quantitative data are presented as means±s.e.m. Differences between two or among more than two groups were evaluated with the two-tailed Student's *t*-test and by one-way analysis of variance (ANOVA) followed by Tukey's post hoc test, respectively. Statistical analysis was performed with Prism software (GraphPad), and *P*<0.05 was considered statistically significant.

### Data availability

The RNA-sequencing data have been deposited in the DNA Data Bank of Japan (DDBJ) under the accession number PRJDB10472.

## Supplementary Material

Supplementary information
