# Supplementary material for: Early manifestations and differential gene expression associated with photoreceptor degeneration in Prom1-deficient retina
Source: Dis Model Mech. 2021 Nov 24;14(11):dmm048962. doi: 10.1242/dmm.048962 (PMC8628633; doi:10.1242/dmm.048962)
Supplement: Supplementary information [file dmm-14-048962-s1.pdf]

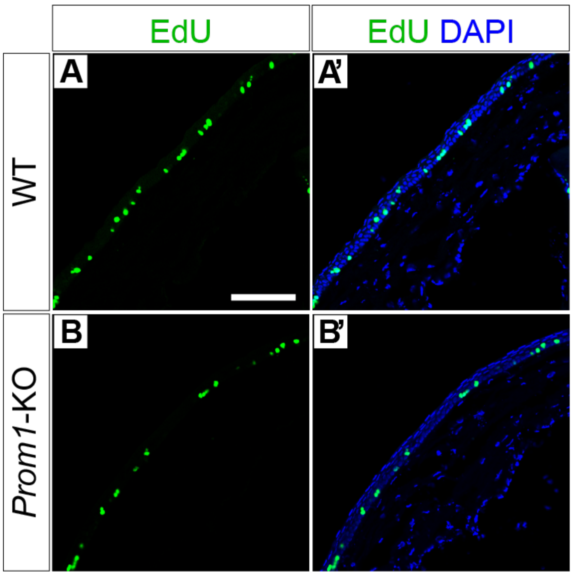

Fig. S1. Intraperitoneally injected EdU reaches the eye. The cornea of the WT (A,A') and *Prom1*-KO (B,B') eyes examined in Fig. 2H-H" and Fig. 2I-I", respectively, was examined for incorporation of EdU-positive cells. Nuclei were stained with DAPI. Scale bar in (A) is 100 µm and applies to all images.

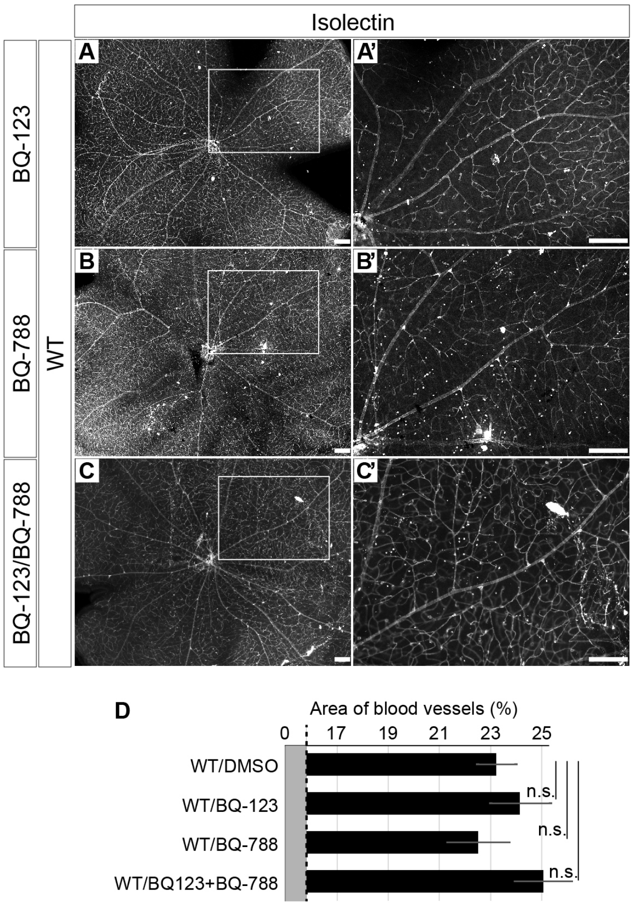

Fig. S2. Endothelin receptor antagonists do not affect retinal vessels of WT mice. (A-C') Isolectin staining of the retina of WT mice treated with BQ-123 (A,A'), BQ-788 (B.B'), or both agents (C,C') as in Fig. 5 and 6. The boxed regions of (A,B,C) are shown at higher magnification in (A',B',C'), respectively. Scale bars, 100 µm. (D) Area of blood vessels measured in images similar to those in (A) to (C'). Data are means ± s.e.m. for five retinas per condition. Note that the data for WT/DMSO are the same as those in Fig. 6F. n.s., not significant (one-way ANOVA followed by Tukey's post hoc test).

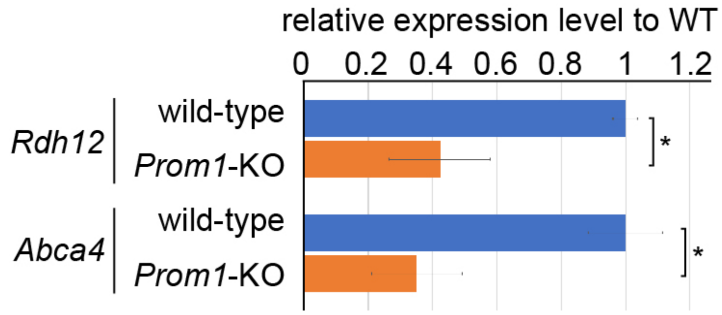

Fig. S3. Expression of *Rdh12* and *Abca4* is down-regulated in the retina of *Prom1*-KO mice at P14. The retina or WT or *Prom1*-KO mice at P14 was subjected to RT-qPCR analysis of *Rdh12* and *Abca4* expression. Data are means ± s.e.m. for three retinas per condition. *$P < 0.05$ (two-tailed Student's *t* test.)

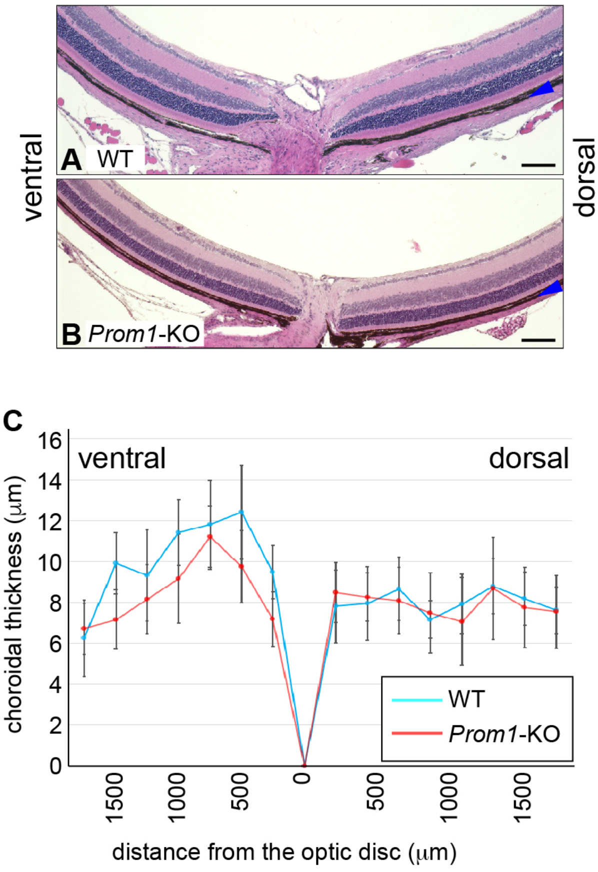

Fig. S4. Choroidal thickness is similar for WT and *Prom1*-KO mice at 4 weeks of age. (A,B) Hematoxylin-eosin staining of the retinas of WT (A) and *Prom1*-KO (B) mice at 4 weeks of age. Blue arrowheads indicate the choroid. Scale bars, 100 µm. (C) Choroidal thickness in sections similar to those in (A) and (B) was measured every 250 µm from the optic disc. Data are means ± s.e.m. for four retinas per condition. No significant difference was apparent between the values for the two genotypes at each distance examined (two-tailed Student's *t* test).

Table S1. RNA-sequencing analysis of the retina of *Prom1*-KO and WT mice at P14.

Click here to download Table S1

Table S2. RNA-sequencing analysis of the retina of *Prom1*-KO and WT mice at P21.

Click here to download Table S2

Table S3. Complete list of pathways affected in the retina of *Prom1*-KO mice at P21 according to GO term analysis.

Click here to download Table S3

Table S4. Sequences of PCR primers used for this study.

Gene	Gene ID	Forward	Reverse
Edn2	13615	GGACATCATCTGGGTGAACA	CAGAAGGTAGCACAGGCAGA
Bcl3	12051	TGCTGAACCTGCCTACTCAC	CAGCGGCTATGTTATTCTGG
Gfap	14580	TGGAGGAGGAGATCCAGTTC	GAGTGCGAATCTCTCTCAGG
Gapdh	14433	ACAACTTTGGCATTGTGGAA	GATGCAGGGATGATGTTCTG
Rdh12	77974	CCAGGAACTCCTACCTGCTG	ACCCACATCCTCTTGCAGTC
Abca4	11304	GGAACTGACAGACATTGCCTGCA	AGAAGTCCACATCGGCATACAGAGT

Disease Models & Mechanisms • Supplementary information
